# The transcriptomic responses of blunt snout bream (*Megalobrama amblycephala*) to acute hypoxia stress alone, and in combination with bortezomib

**DOI:** 10.1186/s12864-022-08399-7

**Published:** 2022-02-25

**Authors:** Shan-Shan Zhao, Xiao-Lei Su, Rong-Jia Pan, Li-Qun Lu, Guo-Dong Zheng, Shu-Ming Zou

**Affiliations:** 1 Genetics and Breeding Center for Blunt Snout Bream, Ministry of Agriculture, Shanghai, 201306 China; 2 Key Laboratory of Freshwater Aquatic Genetic Resources, Ministry of Agriculture, Shanghai, 201306 China; 3 National Demonstration Center for Experimental Fisheries Science Education, Shanghai Ocean University, Shanghai, 201306 China

**Keywords:** Hypoxia, *Megalobrama amblycephala*, Liver, Bortezomib, Transcriptome

## Abstract

**Background:**

Blunt snout bream (*Megalobrama amblycephala*) is sensitive to hypoxia. A new blunt snout bream strain, “Pujiang No.2”, was developed to overcome this shortcoming. As a proteasome inhibitor, bortezomib (PS-341) has been shown to affect the adaptation of cells to a hypoxic environment. In the present study, bortezomib was used to explore the hypoxia adaptation mechanism of “Pujiang No.2”. We examined how acute hypoxia alone (hypoxia-treated, HN: 1.0 mg·L^− 1^), and in combination with bortezomib (hypoxia-bortezomib-treated, HB: Use 1 mg bortezomib for 1 kg fish), impacted the hepatic ultrastructure and transcriptome expression compared to control fish (normoxia-treated, NN).

**Results:**

Hypoxia tolerance was significantly decreased in the bortezomib-treated group (LOE_crit_, loss of equilibrium, 1.11 mg·L^− 1^ and 1.32 mg·L^− 1^) compared to the control group (LOE_crit_, 0.73 mg·L^− 1^ and 0.85 mg·L^− 1^). The HB group had more severe liver injury than the HN group. Specifically, the activities of alanine aminotransferase (ALT) and aspartate aminotransferase (AST) in the HB group (52.16 U/gprot, 32 U/gprot) were significantly (*p* < 0.01) higher than those in the HN group (32.85 U/gprot, 21. 68 U/gprot). In addition, more severe liver damage such as vacuoles, nuclear atrophy, and nuclear lysis were observed in the HB group. RNA-seq was performed on livers from the HN, HB and NN groups. KEGG pathway analysis disclosed that many DEGs (differently expressed genes) were enriched in the HIF-1, FOXO, MAPK, PI3K-Akt and AMPK signaling pathway and their downstream.

**Conclusion:**

We explored the adaptation mechanism of “Pujiang No.2” to hypoxia stress by using bortezomib, and combined with transcriptome analysis, accurately captured the genes related to hypoxia tolerance advantage.

**Supplementary Information:**

The online version contains supplementary material available at 10.1186/s12864-022-08399-7.

## Background

Blunt snout bream (*Megalobrama amblycephala*) is native to the affiliated lakes of the Yangtze River [1, 2]. It is an herbivorous freshwater fish species with a high economic value and high disease resistance in China [3, 4]. Blunt snout bream production output was more than 7.6 × 10 ^5^ tons in 2019 [5]. However, blunt snout bream is a hypoxia-sensitive species, so the large changes in temperature, weather and water quality that decrease the DO (dissolved oxygen) concentration in ponds would cause relatively higher deaths in aquaculture [6]. Therefore, there is a need to breed a new strain with relatively higher hypoxia tolerance. In 2020, “Pujiang No.2”, developed by Shanghai Ocean University, was awarded a new aquatic product variety certificate (Breed Registration Number: GS-01-002-2020). Compared with “Pujiang No.1”, “Pujiang No.2” has a 27% increase in hypoxia tolerance, and the key dissolved oxygen value of body imbalance (LOE_crit_, 25 °C) in the fingerling stage has dropped below 0.90 mg·L^− 1^ [4]. However, the related mechanisms remain unclear. The molecular mechanism of enhanced hypoxia tolerance of “Pujiang No.2” is worth exploring.

Oxygen molecules are indispensable for the normal growth, development and reproduction of organisms [7]. Therefore, metazoans have evolved complex cellular metabolism and physiological systems to maintain oxygen homeostasis [8]. In order to adapt to the hypoxic environment, fish bodies have a series of mediation mechanisms, including changing the respiratory surface area, stimulating angiogenesis, increasing the number of red blood cells or improving the oxygen carrying capacity of hemoglobin, changing the metabolic mode, activating the antioxidant defense system, and changing the expression of related genes [9–12]. Some important factors in the hypoxic signaling pathway strictly regulate this physiological process in fish, and the regulation depends on the expression of genes related to oxygen level [13]. Hypoxia-inducible factor (HIF) is the most critical factor identified in the hypoxic signaling pathway [14]. Bortezomib (PS-341) is an effective proteasome inhibitor. Although bortezomib directly inhibits 26S proteasome, its molecular and cellular effects are profound because proteasome has the necessary cellular homeostasis for protein turnover. Bortezomib indirectly targets mediators of cell cycle progression, regulators of apoptosis, and various transcription factors [15, 16]. Bortezomib is used in the treatment of neoplastic diseases because it affects the adaptation of cells to a hypoxic environment and the cells in the tumor are in a hypoxic environment [17–19]. Bortezomib is considered to be an inhibitor of HIFα, but the molecular mechanism of the inhibitory effect of bortezomib on HIFα is still controversial, and most studies currently focus on HIF1α [17] [20] [21]. HIF1 activates several transcription targets that together promote survival under hypoxic conditions. These enzymes include enzymes involved in glucose uptake and metabolism, carbonic anhydrase IX, which acts in the buffer of glycolytic acid products, erythropoietin, and vascular endothelial growth factor [18]. In recent years, bortezomib has been gradually applied to fish. Stubba et al. (2019) explored the effect of bortezomib on tactile response using a zebrafish embryo model and observed that the tactile response of embryos treated with bortezomib was significantly decreased [22]. Jin et al. (2018) discovered that bortezomib inhibited hypoxia-induced degeneration of ILCM (interlamellar cell mass) in goldfish and affected gill remodeling [23]. These studies will provide the basis for exploring the molecular mechanism of hypoxia resistance in “Pujiang No.2”.

As the hematopoietic organ and the largest gland in fish, the liver plays a vital role in life activities such as material metabolism, detoxification, coagulation and defense. It can best reflect the physiological and pathological state of the body [24]. In addition, liver tissues play essential roles in the body’s adaptation to hypoxia [25]. Therefore, as a critical tissue for studying the hypoxia tolerance mechanism of blunt snout bream, liver tissue has essential significance. RNA-Seq is widely used for transcriptome analysis in plants and animals for the purpose of identifying, analyzing, and quantifying RNA transcripts [26–29]. The transcriptomic analysis enables simultaneous analyses of multiple processes, including protein homeostasis, metabolism and other regulatory cellular processes [30–33]. Recently, liver tissues play vital roles in adaptive hypoxic processes [34, 35]. Therefore, we compared and analyzed liver tissue transcriptome of different groups under hypoxic and normoxic conditions. This study aimed to use bortezomib to explore the hypoxia adaptation mechanism of “Pujiang No.2”, and in combination with transcriptomics and its joint analysis, to accurately capture genes related to hypoxia tolerance advantage.

## Results

### LOE_crit_ of Pujiang no.2

The LOE_crit_ value was used to identify the hypoxic tolerance. Approximately 24 individuals of the bortezomib-treated group and saline-treated group (control group) at 2 different water temperatures were used to determine the LOE_crit_. Our results showed that the LOE_crit_ value varied with bortezomib (Table 1). At 15 °C, compared with the control group (0.73 mg·L^− 1^), the value of LOE_crit_ in the bortezomib-treated group (1.11 mg·L^− 1^) was significantly (*p* < 0.01) increased, indicating that the hypoxia tolerance in the bortezomib-treated group was significantly decreased (Table 1). The same changes were evident in 25 °C, where the LOE_crit_ was 0.85 mg·L^− 1^ in the control group, differing significantly (*p* < 0.01) from the value of the bortezomib-treated group (1.32 mg·L^− 1^) (Table 1). Furthermore, there was significant variation in the LOE_crit_ values between temperatures, with 25 °C showing higher values than 15 °C under the same group.

**Table 1:** The LOE_crit_ at which blunt snout bream lost equilibrium

Groups	LOE_crit_(mg·L^− 1^) 15 °C	LOE_crit_(mg·L^− 1^) 25 °C
Control group	0.73 ± 0.13	0.85 ± 0.11
Bortezomib-treated group	1.11 ± 0.05	1.32 ± 0.16

### Serum enzymes

**Fig. 1:**
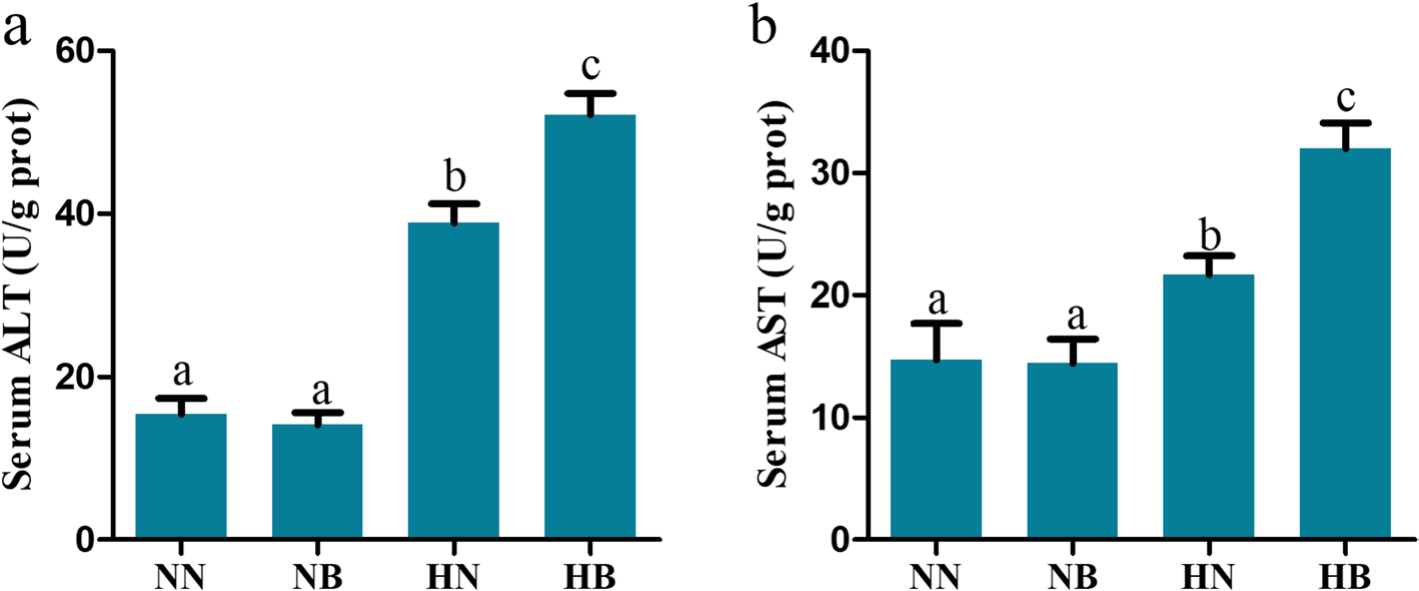
**a** Serum alanine aminotransferase (ALT) and **b** aspertate aminotransferase (AST) levels in normoxia-treated (NN) group, normoxia-bortezomib-treated (NB) group, hypoxia-treated (HN) group and hypoxia-bortezomib-treated (HB) group. The results are given as mean ± SE for separate fish (*n* = 3). Differences among groups were analyzed by unpaired *t*-tests. Columns marked with different letters are significantly different (*p* < 0.01)

To identify liver damage, we determined the activity of ALT (alanine aminotransferase) and AST (aspartate aminotransferase) in serum of blunt snout bream (45 ± 5 g) by commercial kits. Differences in ALT and AST serum levels (Fig. 1a, b) were observed in the groups (HB: hypoxia-bortezomib-treated; HN: hypoxia-treated; NN: normoxia-treated; NB: normoxia-bortezomib-treated) at 15 °C. The activity of ALT and AST in serum of HB group (52.16 U/gprot, 32 U/gprot) was significantly (*p* < 0.01) higher than that of other groups, and the activity of ALT and AST in HN group (32.85 U/gprot, 21.68 U/gprot) was also significantly (*p* < 0.01) higher than that of NB group (14.10 U/gprot, 14.47 U/gprot) and NN group (14.76 U/gprot, 15.41 U/gprot), while the activity of ALT and AST in NB group was similar to that in NN group.

### Histological study of the livers

**Fig. 2:**
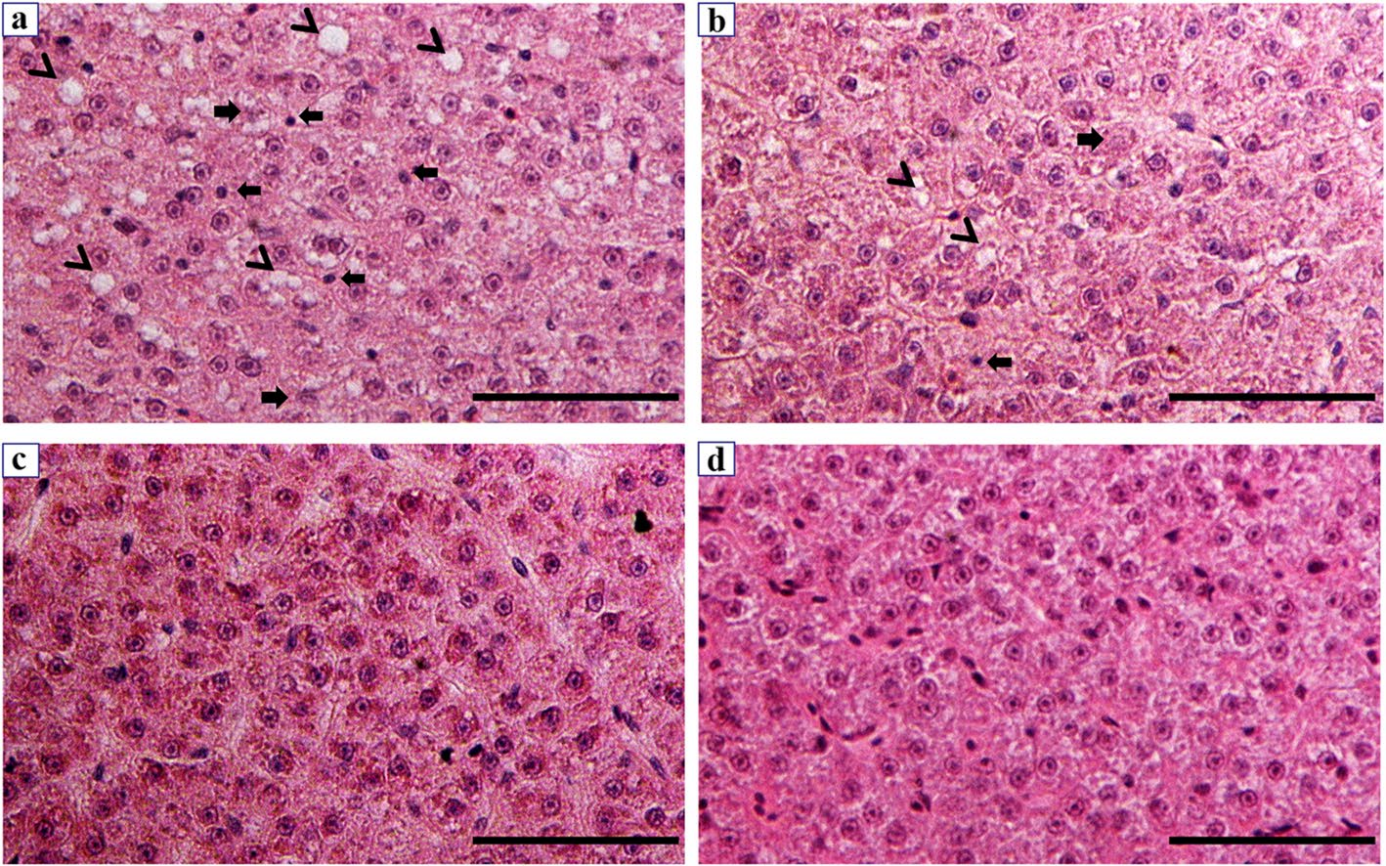
Light microscope micrographs of the liver tissues of “Pujiang No.2” at 15 °C. **a** hypoxia-bortezomib-treated (HB) group, **b** hypoxia-treated (HN) group, **c** normoxia- bortezomib -treated (NB) group, **d** normoxia-treated (NN) groups. The arrows show vacuoles; The left arrows show atrophy of the nucleus; The right arrows indicate nuclear lysis. Scalebars = 50 μm

**Fig. 3:**
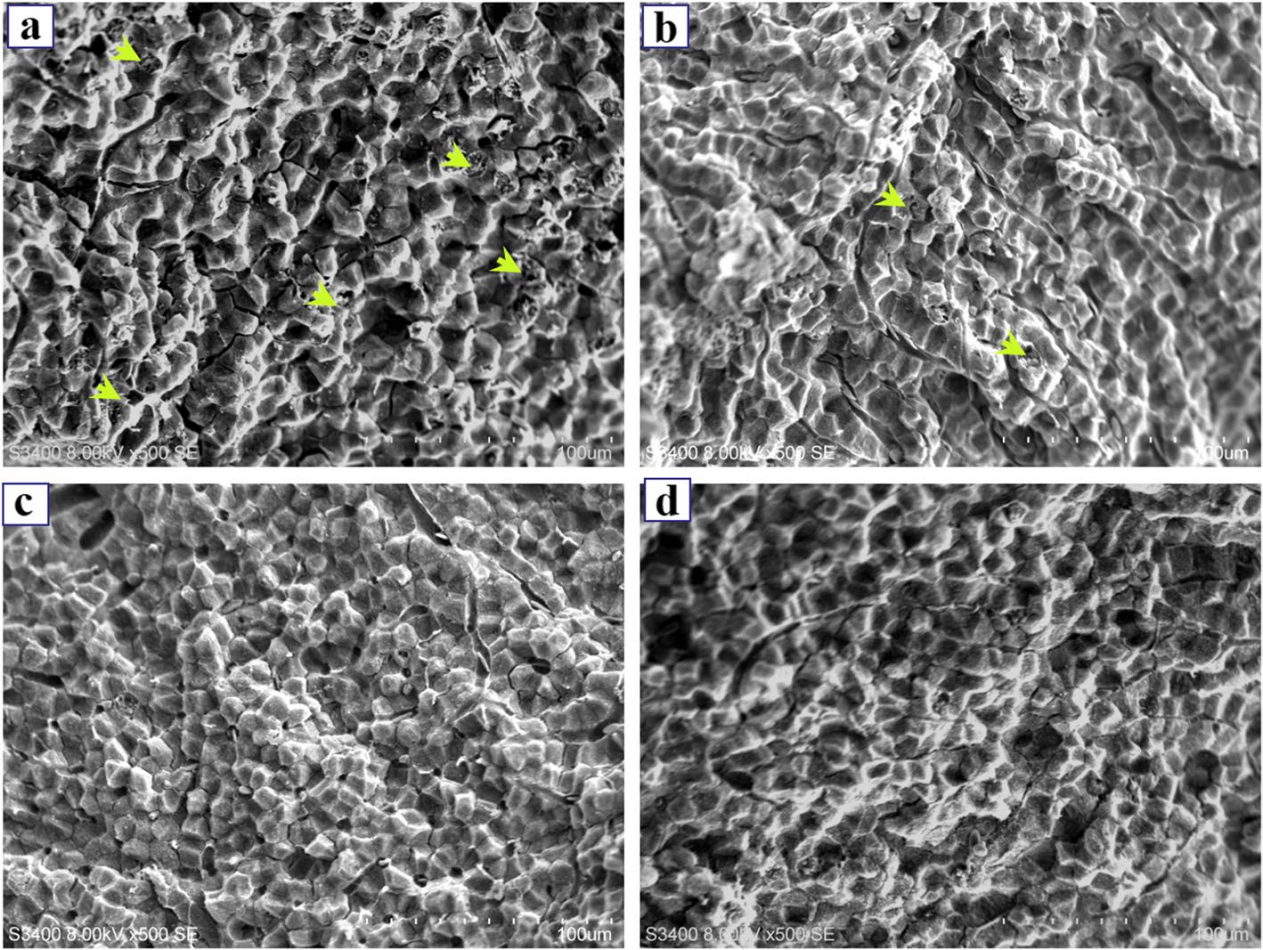
Scanning electron micrographs of the liver tissues of “Pujiang No.2” at 15 °C. **a** hypoxia-bortezomib-treated (HB) group, **b** hypoxia-treated (HN) group, **c** normoxia- bortezomib -treated (NB) group, **d** normoxia-treated (NN) group. The arrows show vacuoles. Scalebars = 100 μm

After fixation, dehydration, transparentizing and waxing of the liver tissue, the samples were embedded in paraffin blocks and cut into 5 μm using a microtome. Then sections are stained and micrographs are taken under an optical microscope. Light microscopy showed that liver damages such as vacuoles, nuclear atrophy and nuclear lysis in the liver tissues of the HN group and HB group (Fig. 2a, b), and the liver damage in HB group was more serious than that in HN group, while there was no obvious change in the liver tissues of NN group and NB group (Fig. 2c, d). More intuitive results were observed by using scanning electron microscopy (Fig. 3). Vacuolization was detected in the liver tissues of HN group and HB group (Fig. 3a, b), while there were no significant changes in the liver tissues of NN group and NB group (Fig. 3c, d). And extensive cavitation was found in HB group.

### Transcriptome assembly and annotation

**Fig. 4:**
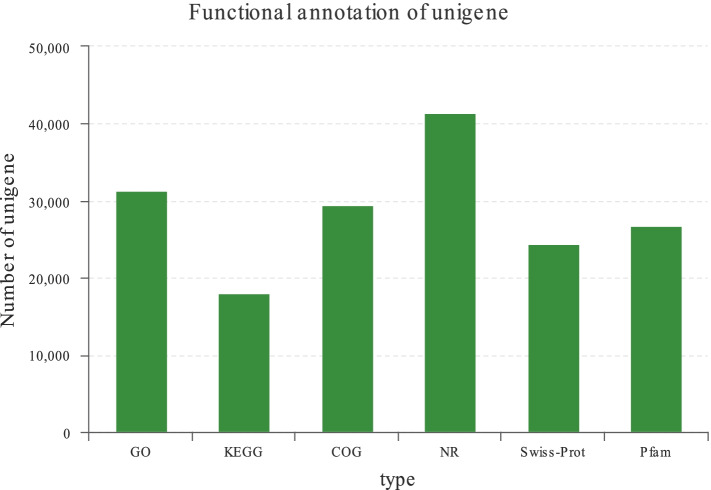
Functional annotation statistics of unigenes. GO, gene ontology; KEGG, kyoto encyclopedia of genes and genomes; COG, clusters of orthologous groups of proteins; NR, nonredundant protein sequence database; Swiss-Prot, annotated protein sequence database; Pfam, protein families

Nine RNA samples (NN, HN, HB, 15 °C) were used for cDNA synthesis and RNA-seq. Transcriptome analysis of nine samples was completed. Over 7.24 GB of raw data for each sample and 451 million reads of the nine samples were obtained (Table 2). A total of 66.78 GB of clean data were obtained (Table 2). The clean data of all samples reached more than 7.09 GB, and the percentage of Q30 bases was more than 95.15% (Table 2). The clean data of all samples were assembled from scratch using Trinity, and the assembly results were optimized and evaluated. The results showed that the number of unigenes obtained by assembly was 103,290, the transcription number was 145,771, and the average length of N50 was 2113 bp (Table 3). All genes and transcripts obtained from transcriptome assembly were compared with six major databases to obtain comprehensive functional information of genes and transcripts and make statistics on the annotation of each database (Fig. 4, Additional file 1).

**Table 2:** The quality of the data

Sample	Raw data				Clean data			
	Raw reads	Raw bases	Q30(%)	GC content (%)	Clean reads	Clean bases	Q30(%)	GC content (%)
HBliver1	48,489,378	7,321,896,078	95.22	48.19	48,180,388	7,199,237,284	95.66	48.11
HBliver2	47,969,462	7,243,388,762	94.87	48.63	47,645,964	7,098,373,630	95.28	48.56
HBliver3	50,753,940	7,663,844,940	95.03	48.38	50,389,498	7,521,948,858	95.52	48.26
HNliver1	50,712,282	7,657,554,582	94.61	48.03	50,337,890	7,505,378,909	95.15	47.91
HNliver2	48,162,454	7,272,530,554	94.93	48.43	47,821,790	7,130,597,017	95.44	48.3
HNliver3	51,403,388	7,761,911,588	94.74	48.47	51,011,834	7,605,795,873	95.29	48.34
NNliver1	49,380,860	7,456,509,860	94.72	48.35	49,030,494	7,333,574,616	95.18	48.25
NNliver2	51,600,530	7,791,680,030	94.82	48.31	51,232,236	7,636,747,653	95.36	48.18
NNliver3	52,374,338	7,908,525,038	94.67	48.34	51,966,604	7,748,338,294	95.21	48.21

**Table 3:** Summary of information regarding the assembly and annotation of the transcriptomes

Type	Unigene	Transcript
Total number	103,290	145,771
Total base	111,998,274	179,479,215
Largest length (bp)	28,684	28,684
Smallest length (bp)	201	201
Average length (bp)	1084.31	1231.24
N50 length (bp)	2113	2427
E90N50 length (bp)	3924	3562
Fragment mapped percent (%)	74.543	85.912
GC percent (%)	42.37	42.95
TransRate score	0.38943	0.4383
BUSCO score	C:96.0% [S:88.3%; D:7.7%]	C:96.0% [S:88.3%; D:7.7%]

### Detection of differentially expressed genes (DEGs)

**Fig. 5:**
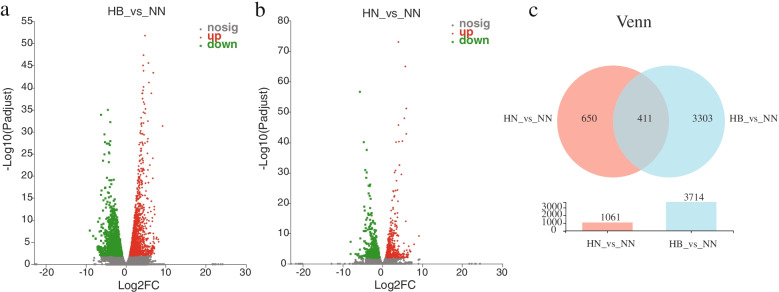
DEGs analysis and volcano plot for (**a**) “HB vs. NN” and (**b**) “HN vs. NN”**.** The x-axis is the value of Log2 (Fold Change), and the y-axis is the value of Log2 (*p*-value). The red dots reveal the up-regulated DEGs, the green reveal the down-regulated DEGs. **c** The overlaps of DEGs between “HN vs. NN” and “HB vs. NN”. Circles of different colors represent the number of unigenes expressed in a group of samples, and the cross area of circles represents the number of unigenes shared by each group. NN, HN and HB denote normoxia-treated group, hypoxia-treated group, hypoxia-bortezomib-treated group

The FPKM (fragments per kilobase million) of each gene in the livers of HB group or HN group were compared with those of NN group, and then the DEGs of “HB vs. NN” and “HN vs. NN” were selected by referring to Zheng et al. (2019) [33]. Volcano plots and heatmap tree were used to describe the number of significantly up and down regulated DEGs of “HB vs. NN” (Fig. 5a, Additional file 2) and “HN vs. NN” (Fig. 5b, Additional file 2). As shown in Fig. 5c, Venn diagram showed that there were 3714 DEGs in “HB vs. NN” and 1061 DEGs in “HN vs. NN”. Among those genes, 411 overlapping DEGs were found in “HB vs. NN” and “HN vs. NN” (Additional file 3).

**Fig. 6:**
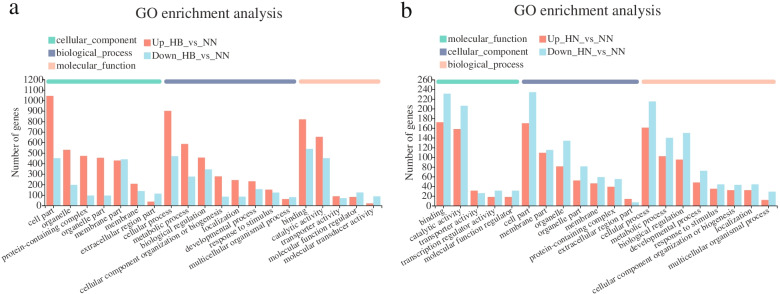
**a** GO enrichment of DEGs of “HB vs. NN”. **b** GO enrichment of DEGs of “HN vs. NN”. NN, HN and HB denote normoxia-treated group, hypoxia-treated group, hypoxia-bortezomib-treated group. Unigenes were annotated in three categories: cellular component, molecular function, and biological process

**Fig. 7:**
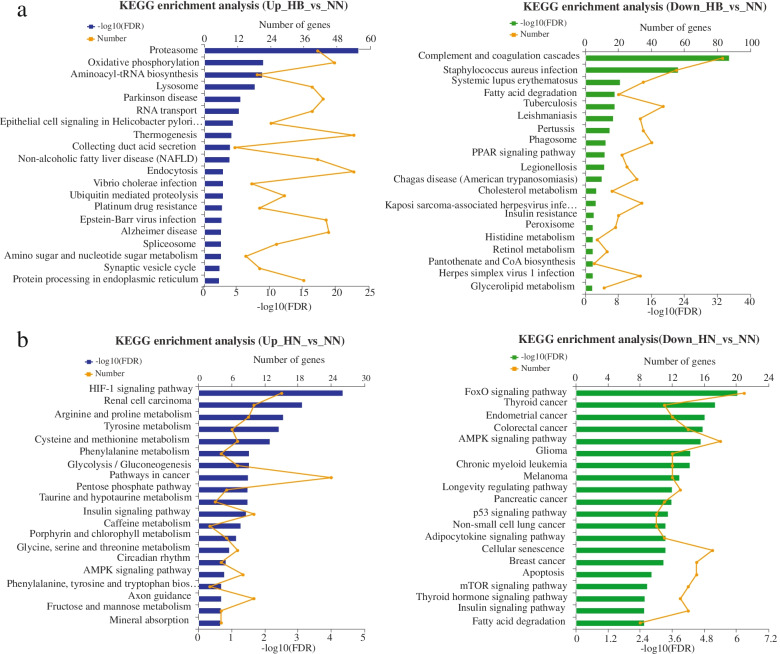
**a** KEGG enrichment of DEGs of “HB vs. NN”. **b** KEGG enrichment of DEGs of “HN vs. NN”. NN, HN and HB denote normoxia-treated group, hypoxia-treated group, hypoxia-bortezomib-treated group. The vertical coordinate indicates KEGG pathway [36], and the upper horizontal coordinate indicates the number of unigenes in the pathway, which corresponds to different points on the polyline. The low abscissa represents that level of significance of enrichment, correspond to the height of the column. Display the enrichment results of Top20

All DEGs were mapped to GO (gene ontology) terms. The livers DEGs of “HB vs. NN” and “HN vs. NN” were enriched into 47 and 45 GO terms (at level 2), respectively. Cellular process, cell part and binding were the three most enriched GO terms in the biological process, cellular component and molecular function categories, respectively (Fig. 6a and b). By analyzing 411 overlapping DEGs in “HB vs. NN” and “HN vs. NN”, cell part, cellular part and binding were the most three enriched GO terms. Among the GO functional categories (detailed GO terms at level 4), some biological processes, including the biosynthetic process, oxidoreductase activity, small molecule metabolic process and oxidation-reduction process, were observed (Table 4). In order to understand the DEGs-enrichment pathways, 1061 DEGs and 3714 DEGs were mapped to reference pathways in the KEGG database [36], respectively. The results showed that 1061 DEGs were mapped to 296 enriched pathways, and 3714 DEGs were mapped to 326 enriched pathways. The first 20 DEGs-enriched KEGG (kyoto encyclopedia of genes and genomes, [36]) pathways were shown in Fig. 7a and b. The up-regulated DEGs in “HB vs. NN” were mainly enriched in the oxidative phosphorylation, thermogenesis and endocytosis pathways; the down-regulated DEGs in “HB vs. NN” were mainly enriched in the complement and coagulation cascades, *staphylococcus aureus* infection, tuberculosis and phagosome pathways (Fig. 7a). And the up-regulated DEGs in “HN vs. NN” were mainly enriched in the pathways in cancer, HIF-1 signaling pathway, AMPK signaling pathway, insulin signaling pathway, and axon guidance pathway; the down-regulated DEGs in “HN vs. NN” were mainly enriched in Foxo signaling pathway, AMPK signaling pathway, insulin signaling pathway, cellular senescence and apoptosis pathway (Fig. 7b). The functions of these enriched pathways were analyzed, which included the catabolism, inflammation, immune, survival, DNA repair and damage prevention, erythropoiesis, angiogenesis, promotion of anaerobic metabolism, cell cycle and oxidative stress resistance pathways. After analyzing these 411 overlapping DEGs, we found that 5 signaling pathways, including HIF-1, FOXO, AMPK, PI3K-Akt, and MAPK signaling pathways, were key pathways for hypoxic tolerance (Table 5). In addition, among the 411 overlapping genes, there were 178 up-regulated DGEs and 233 down-regulated DGEs in “HN vs. NN”; There were 199 up-regulated DGEs and 212 down-regulated DGEs in “HB vs. NN” (Additional files 3, 4 and 5). We analyzed the up-regulated DGEs of 411 overlapping genes, and found that the up-regulated DGEs in “HN vs. NN” were mainly concentrated in Pathways in cancer, HIF-1 signaling pathway, Insulin signaling pathway, Foxo signaling pathway and Alzheimer’s disease; The up-regulated DGEs in “HB vs. NN” were mainly concentrated in the Pathways in cancer, HIF-1 signaling pathway, Alzheimer disease, Insulin signaling pathway, and PI3K-Akt signaling pathway (Additional files 4 and 5). Also, we analyzed the down-regulated DGEs of 411 overlapping genes, and found that the down-regulated DGEs in “HN vs. NN” were mainly concentrated in AMPK signaling pathway, Insulin resistance, MAPK signaling pathway, Pathways in cancer, Fatty acid degradation, Glucagon signaling pathway and Thermogenesis; The down-regulated DGEs in “HB vs. NN” were mainly concentrated in the AMPK signaling pathway, Pathways in cancer, Fatty acid degradation, Insulin resistance, Glucagon signaling pathway and Thermogenesis (Additional files 4 and 5).

**Table 4:** Biological processes related to hypoxia tolerance in blunt snout bream

Number	GO ID	Term Type	Description	*P*value corrected
40	GO:0009058	BP	Biosynthetic process	0.001632
39	GO:0016491	MF	Oxidoreductase activity	0.002165
38	GO:0044281	BP	Small molecule metabolic process	0.001632
16	GO:0044283	BP	Small molecule biosynthetic process	0.004488
15	GO:0016705	MF	Oxidoreductase activity, acting on paired donors, with incorporation or reduction of molecular oxygen	0.003477
13	GO:0055114	BP	Oxidation-reduction process	0.004488

Note: Overlapping DEGs in ‘HB vs. NN’ and ‘HN vs. NN’ were mapped to GO terms. The number of DEGs is greater than 10

**Table 5:** Enriched pathways related to hypoxia tolerance in blunt snout bream

Num	Pathway id	Description	*P*value_corrected	First Category	Second Category
21	map05200	Pathways in cancer	0.092493	Human Diseases	Cancer: overview
14	map04066	HIF-1 signaling pathway	0.00015	Environmental Information Processing	Signal transduction
12	map04152	AMPK signaling pathway	0.003976	Environmental Information Processing	Signal transduction
11	map04922	Glucagon signaling pathway	0.016594	Organismal Systems	Endocrine system
11	map04910	Insulin signaling pathway	0.018591	Organismal Systems	Endocrine system
10	map04360	Axon guidance	0.176007	Organismal Systems	Development and regeneration
10	map04714	Thermogenesis	0.229984	Organismal Systems	Environmental adaptation
10	map04151	PI3K-Akt signaling pathway	0.64764	Environmental Information Processing	Signal transduction
9	map04068	FoxO signaling pathway	0.10064	Environmental Information Processing	Signal transduction
9	map04010	MAPK signaling pathway	0.670013	Environmental Information Processing	Signal transduction

Note: Overlapping DEGs in ‘HB vs. NN’ and ‘HN vs. NN’ were mapped to KEGG pathways. The first 10 DEGs-enriched KEGG pathways were shown

### Qualitative real-time PCR (*q*RT-PCR) validation

**Fig. 8:**
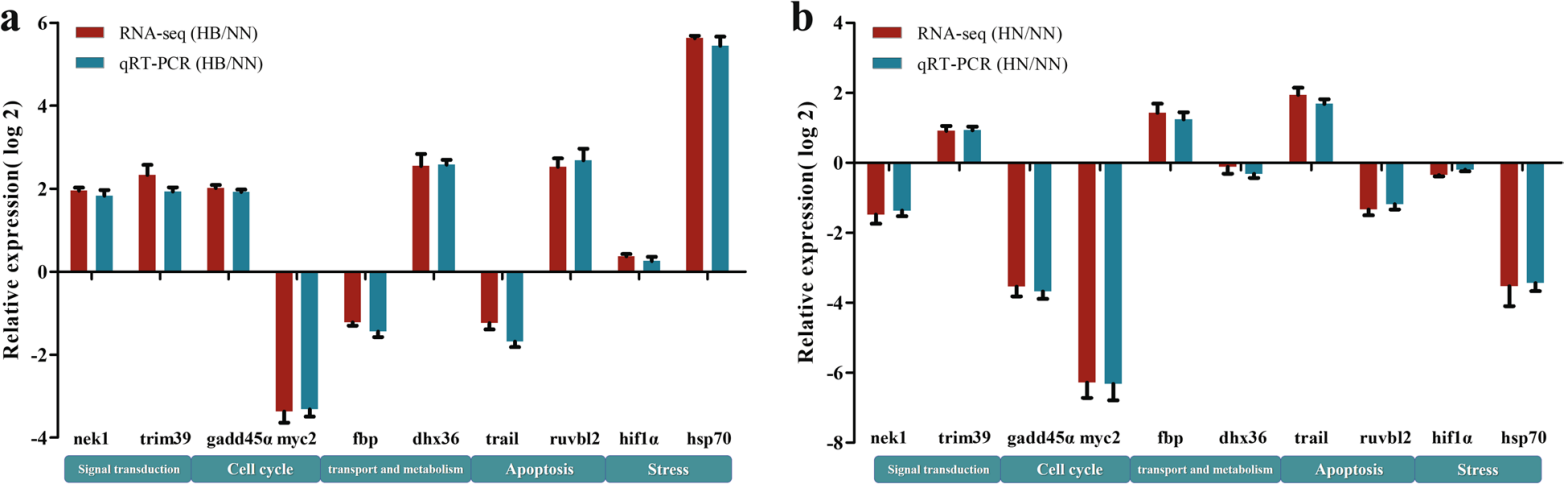
Validation of RNA-seq data of ten DEGs by *q*RT-PCR. 18S was used as an internal control and each value represents average of three separate biological replicates. The results are given as mean ± SE for separate fish (*n* = 3). Differences among groups were analyzed by unpaired *t*-tests

The cDNA was used for *q*RT-PCR analysis using specific primers. Ten DEGs were selected for *q*RT-PCR assay in the livers of these three groups to validate the DEGs in FPKM (Fragments Per Kilobase Million) values. Then *q*RT-PCR results were further compared with the FPKM values generated from RNA-seq. The results showed the data of *q*RT-PCR were consistent with those of RNA-seq (Fig. 8).

## Discussion

Blunt snout bream has always been considered as a hypoxia-sensitive species [2, 4, 37, 38]. The experimental lethal DO value of blunt snout bream was 1.0 ± 0.5 mg·L^− 1^ (water temperature 20-25 °C) [39, 40]. While the experimental lethal DO value of “Pujiang No.2” was dropped below 0.90 mg·L^− 1^ (Water temperature 25 °C) [4]. Therefore, “Pujiang No.2” has strong hypoxia tolerance and extremely high commercial value. However, the molecular mechanism of hypoxia tolerance of “Pujiang No.2” is still unclear, and this is the purpose of our study.

### Bortezomib may affect the hypoxic tolerance in fish

LOE_crit_ was frequently used as an indicator of the hypoxia tolerance of fish species, and time to LOE_crit_ was a standard proxy measure of hypoxia survival in fish, where lower values indicate a higher hypoxia tolerance performance [41–44]. Therefore, in this study, the LOE_crit_ value was used to compare the hypoxic tolerance of the two groups of fish (the control group and the bortezomib-treated group). At 25 °C, the LOE_crit_ of the control group was 0.85 ± 0.11 mg·L^− 1^, similar to the previous study (0.89 ± 0.08 mg·L^− 1^), and which was lower than that of “Pujiang No. 1” at 25 °C (~ 1.03 mg·L^− 1^) [4]. This finding verified that “Pujiang No. 2” had good hypoxia tolerance. What’s more, the LOE_crit_ of the bortezomib-treated group was higher than that of the control group under the same conditions, no matter at 15 °C or 25 °C. It was concluded that bortezomib may affect the hypoxia tolerance of fish.

### Bortezomib may aggravate liver injury in fish under hypoxia stress

The vital amino acid transaminases, alanine aminotransferase (ALT) and aspertate aminotransferase (AST), which are widely distributed in animal mitochondria, play an important role in protein metabolism of the body [45–49]. Enzyme activities of ALT and AST are well-established serum markers of liver function, and their increase may be indexes of liver injury [50–52]. Tabrez et al. (2021) observed high enzyme activities of AST and ALT in *Mystus tengara* which could be due to the lesions in the liver and kidney as a result of bioaccumulation [49]. In Zhang et al. (2020), fermented moringa leaves decreased serum ALT levels and AST levels in gibel carp because it suppressed liver damage [48]. Also, Hajirezaee et al. (2020) observed increases in ALT and AST activities in common carp exposed to TiO2-NPs that indicate metallic-NPs liver damage [53]. In the present study, the activities of ALT and AST in serum of HB (hypoxia-bortezomib-treated) group and HN (hypoxia-treated) group were significantly higher than those in NN (normoxia-treated) group and NB (normoxia-bortezomib-treated) group; and the activities in HB group were significantly higher than those in HN group. These results indicated that hypoxic stress causes liver damage in fish, and that bortezomib treatment in fish under hypoxic conditions maybe exacerbate liver damage.

The liver is a susceptible organ in fish. The liver tissue structure is changed after being damaged, usually resulting in hepatocyte enlargement, vacuolation, and necrosis [54, 55]. Baumann et al. (2020) observed that single cell necrosis of hepatocytes increased with exposure compound concentration in zebrafish [56]. And also, Gong et al. (2020) observed the liver tissue of blunt snout bream from the hypoxia group presented the hepatocellular degenerations [34]. In the present study, the vacuolation of hepatocytes, nuclear atrophy and nuclear lysis was observed in the liver tissues of HB group and HN group by light microscopy. The vacuolation of hepatocytes was observed in the liver tissues of HN group and HB group by scanning electron microscopy. No matter under light microscopy or scanning electron microscopy, more single cell necrosis was observed in liver tissue of HB group. These results also confirmed that bortezomib may aggravate liver injury in fish under hypoxic stress.

### Biological processes related to hypoxia tolerance in fish

Significant changes were found in the transcription of genes involved in several biological processes by analysis of GO functional enrichment. For example, the “biosynthetic process”, “oxidoreductase activity”, “small molecule metabolic process”, “small molecule biosynthetic process”, and “oxidation-reduction process”. The “oxidoreductase activity” and “oxidation-reduction process” associated with oxidative stress and adaptation to environmental stress, had been widely studied [34, 35]. We further analyzed the DEGs and found that Fructose-1,6-bisphosphatase (fbp) and acetyl-CoA carboxylase (acc) were more highly expressed in fish and belonged to the subcategory “small molecule metabolic process”, “small molecule biosynthetic process” and “biosynthetic process”. Fbp is considered to be one of the flux-regulating enzymes of the gluconeogenic pathway [57]. Acc is a key metabolite in the regulation of energy homeostasis [58]. Therefore, DEGs in the “small molecule metabolic process”, “small molecule biosynthetic process” and “biosynthetic process” might be associated with hypoxia adaptation in fish.

### Pathways related to hypoxia tolerance in fish

The HIF-1 signaling pathway played important roles in oxygen homeostasis. HIFs are major regulators of cellular response to reduced oxygen levels and regulate the expression of genes related to oxygen level dependence [14]. HIF1α plays a key role in the conversion between aerobic and anaerobic respiration [59]. In the present study, the expression of key gene (*egln*) of HIF-1 signaling pathway was significantly up-regulated in HN and HB, compared with that in NN group. In fish, expression of the *egln* gene under hypoxic conditions is responsive to the HIF signaling pathway that mediates oxygen-dependent degradation of hypoxia-inducible factor subunits to regulate oxygen balance [60]. In HN group, the expression of downstream genes (*epo*, *vegf*, *homox1*, *cnkn1*) of HIF-1 signal pathway increased significantly. *Epo* gene is involved in erythopoesis process; *Vegf* gene participates in angiogenesis process; *Homox1* gene is involved in vascular tone process; And *Cnkn1* gene is involved in the process of regulating proliferation and apoptosis. In the HB group, the expression of downstream genes (*tnfrsf3*, *homox1*, *angpt*, *slc2a*, *hk*, *gapdh*, *eno*, *pfkfb3*) of HIF-1 signaling pathway was significantly increased. *Tnfrsf3* gene is involved in inflammation process; *Angpt* gene is involved in angiogenesis process; Genes (*slc2a*, *hk, gapdh*, *eno*, *pfkfb3*) are involved in promote anaerobic metabolism process. Bortezomib is considered to be an inhibitor of HIFα [21]. In our study, expression of *hif1α* was slightly increased in the HN group and slightly decreased in the HB group when compared with the NN group. Our results are consistent with previous studies. These results indicate that these DEGs might be related to the higher ability of “Pujiang No. 2” to resist hypoxia.

The FOXO signaling pathway was involved in the cell apoptosis, autophagy, DNA damage, angiogenesis, oxidative stress resistance, glucose metabolism, tumorigenesis and glycolysis/gluconeogenesis [61–65]. Kops et al. (2002) concluded that *foxo3* protected quiescent cells from oxidative stress [66]. Members (*foxo1*, *foxo3*, *foxo4*, *foxo6*) of the FOXO signaling pathway were significantly downregulated in HN group and HB group compared to NN group in the present study. In addition, the downstream genes (*bcl2l11*, *bnip3*, *gabarap, pepck*, *g6pc*, *gadd45α*, *atm*, *ccnb1/2*, *ccng2*, *plk* and *cdkn1a*) were more highly expressed in HN group. The downstream genes (*bcl2l11*, *bnip3* and *gabarap*) were involved in the apoptosis and autophagy pathways, which was a positive respose to alleviating liver injury.; the downstream genes (*pepck* and *g6pc*) were involved in glycolysis/gluconeogenesis pathway [67]; the downstream genes (*gadd45α* and *atm*) were involved in the oxidative stress resistance and DNA repair process; the downstream genes (*cdkn1a*, *ccng2*, *ccnb1/2*, *plk*, and *gadd45α*) were involved in cell cycle regulation. Glycogen in the liver could be consumed by anaerobic glycolysis to maintain ATP levels under hypoxic conditions [34]. The activation of the cell cycle regulation pathway, oxidative stress resistance and DNA repair pathway in fish was also a positive pathway against hypoxia stress. However, in the HB group, only a few genes (*plk*, *gabarap* and *ccng2*) had increased expression, which may explain why the HB group was not resistant to hypoxia. These results indicate that these DEGs might be related to the higher ability of “Pujiang No. 2” to resist hypoxia. And it was speculated that after acute hypoxia stress, FOXO pathway was activated and then the body could save itself through the autophagy and apoptosis pathways, and then the body could save itself through the glycolysis/gluconeogenesis pathway, the oxidative stress resistance and DNA repair pathway, the cell cycle regulation pathway, the glycolysis/gluconeogenesis pathway, the oxidative stress resistance and DNA repair pathway, and the cell cycle regulation pathway.

The MAPK family is an important signaling molecule. A variety of intracellular and extracellular stimuli, including growth factors, hormones, oxidative stress, and endoplasmic reticulum stress, can regulate various cellular activities by activating the MAPK signaling pathway [68]. Ramkumar et al. (2018) stimulated that p38 MAPK activation with hypoxia and acted on downstream MAP4. And after MAP4 activation, tubulin was induced to be unstabilized, which made endothelial cell proliferation and migration active and promoted pulmonary neovascularization [69]. In the previous studies, the hypoxic response was shown to be closely related to the oxidative phosphorylation by MAPK signaling pathway [25]. In our research, members (*dusp*, *mknk* and *myc*) of the MAPK signaling pathway were significantly upregulated in HB group and HN group, activation of these genes may be associated with hypoxia tolerance. What’s more, activation of MAPK signaling pathway could affect HIF-1 signaling pathway and FOXO signaling pathway. These results indicated that these DEGs might be related to the high hypoxia tolerance of “Pujiang No. 2”.

The PI3K-Akt signaling pathway induces cell proliferation and endothelial cell differentiation, inhibits apoptosis, promotes epithelial cell mesenchymal transition, and induces neovascularization [70–72]. In Sun et al. (2016), upregulation of genes expression of PI3K-AKT signaling pathway members by hypoxic stress [73]. Also, Yang et al. (2018) found that some genes of PI3K-Akt signaling pathway were expressed at higher levels of channel catfish in the hypoxia-treated group than in the control group [32]. In the present study, members (*ntrk*, *pi3kr* and *pi3kc*) of PI3K-Akt signaling pathway were significantly upregulated in HN group and HB group compared to NN. Activation of PI3K-Akt signaling pathway affects HIF-1 signaling pathway and FOXO signaling pathway. The downstream genes (*cys*, *pepck*, *myc*, *ccnd*, *bcl2l11* and *mcl1*) of PI3K-Akt signaling pathway were involved in the metabolism and cell survival process which more highly expressed in HN. While downstream genes (*g6pc*, *myc*, *bcl2l1*, *mcl1* and *tp53*) were also involved in the metabolism and cell survival process which more highly expressed in HB. These results indicate that these DEGs might be related to the higher ability of “Pujiang No. 2” to resist hypoxia.

AMP-activated protein kinase (AMPK) is a key regulator of intracellular homeostasis and plays an active role in regulating energy metabolism and controlling inflammation [74, 75]. AMPK is activated during adaptation to hypoxic and ischemic stress, resulting in beneficial effects on the organism [76]. When tissues are hypoxic, cells are activated by AMPK because of a decrease in metabolically stimulated energy, i.e., an increase in the AMP/ATP ratio. AMPK then increases ATP production from by upregulating catabolic-related genes [74, 77]. Jibb and Richards (2008) found that a ~ 5.5-fold increase in AMPK activity in hypoxia-tolerant goldfish after 0.5 h of exposure to severe hypoxia [78]. In Yang et al. (2019), Hypoxia induced the expression of *AMPKα* in largemouth bass [79]. In the present study, members (*ppp2*, *prka* and *eef2k*) of the AMPK signaling pathway were significantly upregulated in HN group and HB group compared to NN group, and activation of these genes might affect hypoxia tolerance.

## Conclusions

In this study, we found that bortezomib affects hypoxic tolerance in fish. We performed RNA-seq of the livers of hypoxia-treated (HN) group, hypoxia-bortezomib-treated (HB) group and normoxia-treated (NN) group. RNA-seq was performed on livers from the HN, HB and NN groups. KEGG pathway analysis disclosed that many DEGs (differently expressed genes) were enriched in the HIF-1, FOXO, MAPK, PI3K-Akt and AMPK signaling pathway and their downstream. In addition, we found that treatment with bortezomib might aggravate the hypoxic stress response of “Pujiang No.2”. The results of our study indicated the genes and signaling pathways related to the molecular mechanism of hypoxia tolerance in “Pujiang No.2”, which were of great significance for fish genetics and breeding.

## Materials and methods

### Experimental fish

Blunt snout bream specimens were obtained from the Bream Genetics and Breeding Center (BGBC) of Shanghai Ocean University, Shanghai, China. Specimens that belonged to “Pujiang No.2” breed were used. The new variety, “Pujiang No.2”, is developed by taking the wild blunt snout bream collected in Poyang Lake as the primary population, taking the growth traits as the main breeding index, and adopting the population breeding technology supplemented by the hypoxia stress technology through four successive generations. A total of 200 juvenile fish (45 ± 5 g) were acclimatized in indoor tanks for 2 weeks with 2 different water temperature (i.e., 15 ± 0.1 °C, 25 ± 0.1 °C), respectively. The water temperature in the tanks were controlled by the element. One hundred juvenile fish were transferred from the outdoor cement pool to the indoor tank (water temperature 15 ± 0.1 °C), and the same token, one hundred juvenile fish were transferred from the outdoor cement pool to another indoor tank (water temperature 25 ± 0.1 °C) for acclimation for 2 weeks. Fifty juvenile fish at the same water temperature were intraperitoneally injected with 0.1 mL sterile saline alone or bortezomib (1 mg·kg^− 1^, Selleck) in 0.1 mL sterile saline. Usage and dosage of bortezomib determined by reference to Jin et al. (2019) [23]. After injection, all juvenile fish were returned to the tanks to for holding 20 h by marking the difference.

### Oxygen tension threshold for loss of equilibrium

The DO concentrations in the waters described in last section were 8.6 ± 0.3 mg·L^− 1^. And then, determination of the LOE_crit_ was initiated. The glass tanks and the methods for treatments were performed as previously described by Wu et al. (2020) [4], and the [O_2_] was decreased in a stepwise manner (decreased over 30 min and then held at the new level for 30 min), in increments of 0.5 mg·L^− 1^, to a final level of 0 mg·L^− 1^. Oxygen levels were monitored using oxygen electrodes (YSI, ProODO, Germany). When a fish showed loss of equilibrium, the [O_2_] and time were recorded. The LOEcrit was calculated for fish using Brett’s equation [80]:

$${\mathrm{LOE}}_{crit}={\left[{\mathrm{O}}_2\right]}_{2i}-\left(\frac{t_i}{t_{ii}}\right){\left[{\mathrm{O}}_2\right]}_{2 ii}$$

where [O_2_]_2i_ is the lowest level of O_2_ at which the fish could maintain equilibrium for the full duration; [O_2_]_2ii_ is the decrease in O_2_ tension at each increment (0.5 mg·L^− 1^); t_i_ is the time required for the fish to lose equilibrium at the final [O_2_]_2_; and t_ii_ is the time held at each [O_2_]_2_. Approximately 24 individuals of the bortezomib-treated group and saline-treated group (control group) at 2 different water temperatures were used to determine the LOE_crit_. This experiment for LOE_crit_ was repeated 5 times.

### Hypoxia treatment

Twelve juvenile fish from the bortezomib-treated group (15 °C) and 12 fish from saline-treated group (15 °C) were kept in 25 L glass tanks for 6 h filled with dechlorinated tap water bubbled with N_2_ gas and air ((hypoxia-treated, HN; hypoxia-bortezomib-treated, HB). The bubbling rate of N_2_ and air were controlled to achieve an DO of 1.0 mg·L^− 1^. Similarly, 12 juvenile fish from the bortezomib-treated group (15 °C) and 12 juvenile fish from saline-treated group (15 °C) were kept with an DO of 8.6 mg·L^− 1^ in 25 L glass tanks for 6 h (normoxia-treated, NN; normoxia -bortezomib-treated, NB). At the end of experiments, the fish were anesthetized using MS-222 (100 mg·L^− 1^) and then sampled from each group after hypoxia or normoxia treatment. This part of the experiment was repeated 3 times.

### Enzyme activity assays

After the hypoxia or normoxia treatment described in the last section, blood was taken from the caudal vein with a 1 mL syringe. And once coagulation took place in 1.5 mL tubes for 4 h, blood was centrifuged for 15 min at 5000 rpm. Serum was collected and analyzed for alanine aminotransferase (ALT) and aspartate aminotransferase (AST). The activity of enzymes was detected using commercial kits (Alanine aminotransferase Assay Kit and Aspartate aminotransferase Assay Kit) produced by Jiancheng Bioengineering Institute (Nanjing, China). This part of the experiment was repeated 3 times.

### Light microscopy and scanning electron microscopy analyses

After the hypoxia or normoxia treatment, liver tissues from 3 random individuals of each group were used as samples. The samples were processed in the manner previously described [4], i.e., fixation, dehydration, transparentizing and waxing of the liver tissue. Then, the samples were embedded in paraffin blocks and they were taken using a microtome (RM2125RT; Leica, Germany) into 5 μm slices. Finally, the slices were stained, and microphotographs were taken under a light microscope (Eclipse 80i; Nikon, Japan). For the scanning electron microscopy (SEM) analysis, the livers were processed in the manner previously described [4]. Simply put, liver tissues were treated with fixation, dehydration, and drying. Finally, the ionocytes were examined and photographed with a Hitachi S-3400 N scanning electron microscope.

### RNA extraction, library construction and sequencing

Total RNA of the liver samples was extracted with RNAiso Plus (Takara, Japan) and purified with RNeasy Mini kit (Qiagen, USA). The purity and concentration of RNA was determined by NanoDrop2000 and the integrity of RNA was determined by Agilent 2100 Bioanalyzer (Agilent Technologies, USA). Nine RNA samples (NN, HN, HB, 15 °C) were used for cDNA synthesis and RNA-seq. Before library construction, poly (A) + mRNA was isolated by Magnetic Oligo-dT beads from Invitrogen (USA), and cDNA libraries were constructed and sequenced by Illumina Novaseq 6000 platform (Majorbio Biotech Co., Ltd., China). Before sequencing, the DNA libraries were quantified by using TBS380 micro fluorometer with Picogreen® reagent (Invitrogen, USA). Clusters were generated by bridge PCR amplification on Illumina Bot. Illumina Novaseq 6000 sequencer for high-throughput sequencing.

### De novo assembly and functional annotation of unigenes

The raw sequencing data were controlled using the previously described methods to obtain high quality data [33]. All clean reads were then assembled using the de novo assembly program Trinity. All the obtained transcripts were compared with NR [non-redundant protein, ftp://ftp.ncbi.nlm.nih.gov/blast/db/], Swiss-Prot [http://www.uniprot.org/], Pfam [http://pfam.xfam.org/], COG [Clusters of Orthologous Groups of proteins, http://www.ncbi.nlm.nih.gov/COG/], GO [Gene Ontology, http://www.geneontology.org/], and KEGG [Kyoto Encyclopedia of Genes and Genomes, http://www.genome.jp/kegg/] databases to obtain annotation information in each database, and the annotation status in each database was counted. In addition, functional annotation including biological process, molecular function and cellular component for GO terms were analyzed by using BLAST2GO software to understand the distribution of gene functions [81].

### Comparative expression analysis

The RSEM Software [http://deweylab.biostat.wisc.edu/rsem/] was used to conduct quantitative analysis on the expression level of genes or transcripts and reveal the regulatory mechanism of genes based on sequence function information [82]. And DESeq2 [http://bioconductor.org/packages/stats/bioc/DESeq2/] was used for differential expression analysis of genes or transcripts between samples, thereby revealing the functions of the differential genes or transcripts [83]. And then, the enrichment analysis of GO and KEGG pathways was carried out by Goatools software (version 0.4.7) and KOBAS 2.0 software [83].

### Qualitative real-time PCR (*q*RT-PCR)

After the total RNA was extracted from the livers, the cDNA was synthesized by the PrimeScript RT reagent Kit (Takara, Janpan). It was then used for *q*RT-PCR analysis using specific primers (Table 6). Instrument, reagents, method as previously described [3]. The housekeeping gene 18S was used as the control, which had been proved to be stable between the comparison groups. All experiments were performed for three replicates.

**Table 6:** Primer sequences used in this study

Primer name	Primer sequence (5′–3′)	Assay technique
*GADD45α*-F1	CACATGCATCCACATGGAAA	qRT-PCR
*GADD45α*-R1	TTCTCATCGTTCTGGAAGGTTG	
*NEK1*-F1	GAGCAGTCTGAGCCTGAGGA	qRT-PCR
*NEK1*-R1	GACCAGCTGCCTCTTCATCG	
*MYC2*-F1	GCTCTCAAGCGCTGTCACTT	qRT-PCR
*MYC2*-R1	GTCCTGAACTCTGCGTGCTT	
*HSP70*-F1	GACCCAGACCTTCACCACCT	qRT-PCR
*HSP70*-R1	GATTCCGTTGGCGTCGATGT	
*DHX36*-F1	CAGAGCCTCACATCCCTCCA	qRT-PCR
*DHX36*-R1	GTCCGCACAACATCCTGGAA	
*Hif1αb*-F1	ACACAGAGCGCAGCTTCTTC	qRT-PCR
*Hif1αb*-R1	GACATGACCAGCGCAGTGAA	
*MSH2*-F1	AATTGGGCAGAGGCACATCC	qRT-PCR
*MSH2*-R1	TGACGTGGAGGTTACGGACA	
*RUVBL2*-F1	GATGCCATGGGCTCACAGAC	qRT-PCR
*RUVBL2*-R1	TGCTCCCGAACCTCTGACTT	
*TRAIL*-F1	GGTGCAAACATTTGGCGCTT	qRT-PCR
*TRAIL*-R1	ACGCACCATGTCCAAACAACT	
*TRIM39*-F1	ACACACCAACCCAGAGCACT	qRT-PCR
*TRIM39*-R1	GAAAGTCTTTTAGCTTCCACTGTG	
18S-F	ACCGCAGCTAGGAATAATGG	qRT-PCR
18S-R	GGTCGGAACTACGACGGTAT	

## Supplementary Information

**Additional file 1.**

**Additional file 2.**

**Additional file 3.**

**Additional file 4.**

**Additional file 5.**

**Additional file 6.**

## Data Availability

Clean data supporting the results of this article were uploaded to the NCBI Sequence Read Archive (SRA) website under accession number PRJNA723430. (See Additional file 6 for details on abbreviated genes).

## Abbreviations

HB: Hypoxia-bortezomib-treated
HN: Hypoxia-treated
NN: Normoxia-treated
NB: Normoxia-bortezomib-treated
LOE: Loss of equilibrium
DO: Dissolved oxygen
ALT: Alanine aminotransferase
AST: Aspartate aminotransferase
DEGs: Differently expressed genes
HIF: Hypoxia-inducible factor
ILCM: Interlamellar r cell mass
NR: Non-redundant protein
COG: Clusters of orthologous groups of proteins
GO: Gene ontology
KEGG: Kyoto encyclopedia of genes and genomes
*q*RT-PCR: Qualitative real-time PCR
FPKM: Fragments Per Kilobase Million
