# Supplementary material for: The transcriptomic responses of blunt snout bream (Megalobrama amblycephala) to acute hypoxia stress alone, and in combination with bortezomib
Source: BMC Genomics. 2022 Feb 25;23:162. doi: 10.1186/s12864-022-08399-7 (PMC8876555; doi:10.1186/s12864-022-08399-7)
Supplement: Supplementary file 1 — Additional file 1. [file 12864_2022_8399_MOESM1_ESM.pdf]

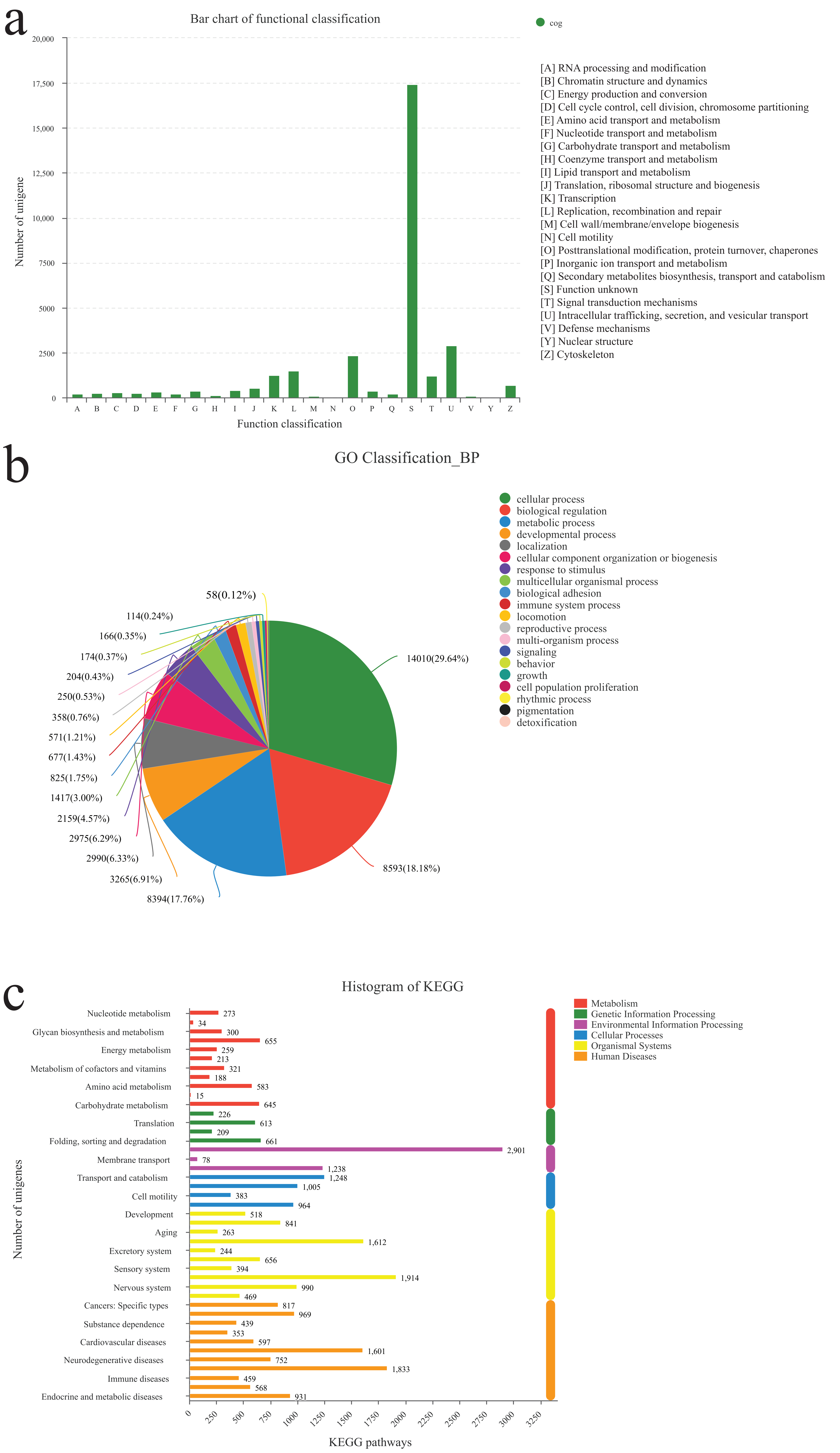

Additional file 1. Functional annotation statistics of unigenes corresponding to the database in "Pujiang No.2". (**a**) COG functional classification of unigenes. (**b**) GO classification of unigenes. (**c**) Enriched pathways associated with unigenes.
