# Supplementary material for: The transcriptomic responses of blunt snout bream (Megalobrama amblycephala) to acute hypoxia stress alone, and in combination with bortezomib
Source: BMC Genomics. 2022 Feb 25;23:162. doi: 10.1186/s12864-022-08399-7 (PMC8876555; doi:10.1186/s12864-022-08399-7)
Supplement: Supplementary file 2 — Additional file 2. [file 12864_2022_8399_MOESM2_ESM.pdf]

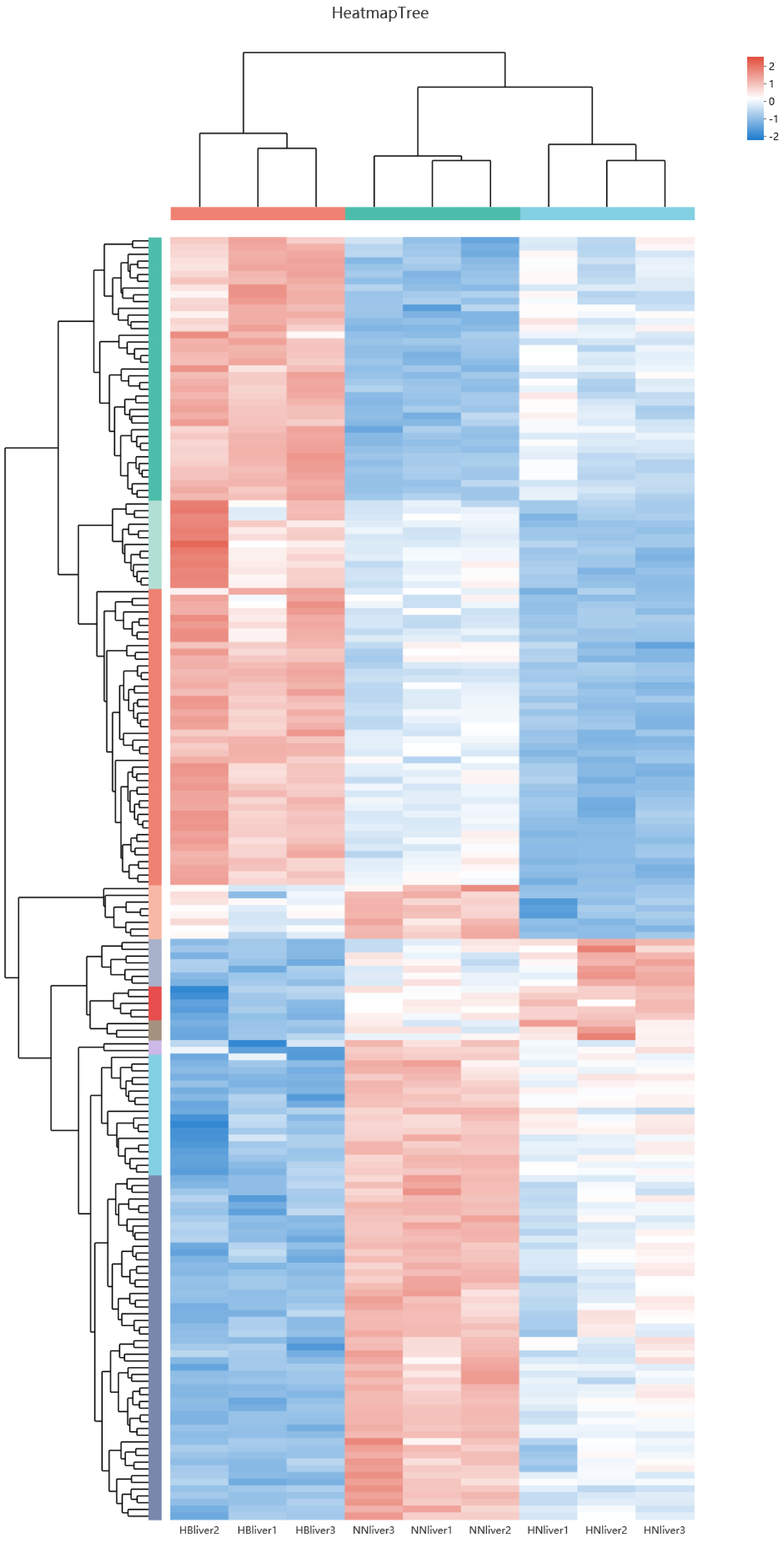

Additional file 2. Hierarchical cluster analysis of DEGs in the blunt snout bream. The colour key represents FPKM normalized log2 transformed counts in livers of three groups (NN, HN and HB groups). Changes in expression levels are shown using colour scales with saturation at > 2-fold changes. Red and Blue gradients indicate an increase and decrease of DEGs, respectively.
